# A Quantized Convolutional Neural Network Implemented With Memristor for Image Denoising and Recognition

**DOI:** 10.3389/fnins.2021.717222

**Published:** 2021-09-16

**Authors:** Yuejun Zhang, Zhixin Wu, Shuzhi Liu, Zhecheng Guo, Qilai Chen, Pingqi Gao, Pengjun Wang, Gang Liu

**Affiliations:** ^1^Faculty of Electrical Engineering and Computer Science, Ningbo University, Ningbo, China; ^2^Department of Micro/Nano Electronics, School of Electronic Information and Electrical Engineering, Shanghai Jiao Tong University, Shanghai, China; ^3^School of Materials, Sun Yat-sen University, Guangzhou, China; ^4^College of Mathematics, Physics, and Electronic Information Engineering, Wenzhou University, Wenzhou, China

**Keywords:** memristor, conductance fine-tuning, synaptic plasticity, convolutional neural network, image denoising

## Abstract

The interference of noise will cause the degradation of image quality, which can have a negative impact on the subsequent image processing and visual effect. Although the existing image denoising algorithms are relatively perfect, their computational efficiency is restricted by the performance of the computer, and the computational process consumes a lot of energy. In this paper, we propose a method for image denoising and recognition based on multi-conductance states of memristor devices. By regulating the evolution of Pt/ZnO/Pt memristor wires, 26 continuous conductance states were obtained. The image feature preservation and noise reduction are realized *via* the mapping between the conductance state and the image pixel. Furthermore, weight quantization of convolutional neural network is realized based on multi-conductance states. The simulation results show the feasibility of CNN for image denoising and recognition based on multi-conductance states. This method has a certain guiding significance for the construction of high-performance image noise reduction hardware system.

## Introduction

Image denoising plays an important role in visual processing. In the process of image perception, noise destruction will inevitably occur, which will seriously reduce the visual quality of the acquired image and further reduce the accuracy of image recognition (Andrews and Hunt, 1977; Chatterjee and Milanfar, 2010). In addition to the traditional algorithm of designing filter for image denoising (Ullah and Halim, 2021; Upadhyay et al., 2021), the current popular and effective algorithm is the deep learning algorithm for image denoising and feature extraction (Zhang et al., 2017; Tian et al., 2020a,b; Wang et al., 2020). However, the above algorithms all run on the von Neumann computer architecture; thus, a large amount of energy consumption will be required during the calculation process, while its computational efficiency is limited (Chen et al., 2018; Xia and Yang, 2019; Zhang W. Q. et al., 2020). Similar to how the human brain transmits and processes information through a large number of interconnected neurons and synapses, neuromorphic computing approaches based on new solid-state electronics have demonstrated high efficiency, low power consumption, and parallel processing capabilities for big data analysis tasks (Chua, 1971; Akinaga and Shima, 2010). Among them, the resistance switch memristor is considered the best candidate for the post-Moore era due to its simple device structure and the very large-scale integration capability of the crossbar array. In addition, studies have shown that memristor-based in-memory computation is compatible with complementary metal-oxide-semiconductor (CMOS) technology (Merced-Grafals et al., 2016; Li et al., 2018).

Typically, the memristors consisted of two electrode layers (top and bottom electrodes) and a dielectric layer (Sawa, 2008; Brivio et al., 2018), and when the scanning or pulse voltage was applied by the top electrode through the dielectric layer to a bottom electrode, they displayed a continuous multi-conductance state changes, similar to the regulation of the synaptic connection strength, which is called synaptic plasticity (Zhou et al., 2019; Gao et al., 2021). At present, a number of studies have shown that such characteristics, which are similar to the neuronal structure and the transmission mode of neurotransmitters, have great advantages in realizing multi-valued information storage and constructing memristor neural networks (Zhang et al., 2019; Yao et al., 2020; Kimura et al., 2021). The first step of the neural network in image recognition is to transform the input image into a gray matrix with pixel value between 0 and 255. In this process, the input image with noise will directly change the distribution of pixel value in the gray matrix, thus, affecting the subsequent iterative calculation results of the network. By using the conductance property of memristor to transform the pixel value into several discrete conductance states, the influence of the change in pixel value distribution can be reduced by quantifying the image information. At the same time, based on the characteristics of multi-conductance continuous change in memristor, the mapping relationship between the weight of neural network and the conductance of memristor can be constructed to realize the memristor neural network (Ma et al., 2020; Sun et al., 2021).

In this paper, we report an effective method for feature preservation and image denoising based on the multi-conductance switching property of memristor devices. Meanwhile, the mapping relationship between the weight of neural network and the conductance of memristors is constructed by using the multi-conductance properties. Finally, a memristor CNN, which can be used for image denoising and recognition, is realized. The additive white Gaussian noise (AWGN) was used to introduce noise to handwritten letters as input, and the input images were quantized and denoised using conductance curves measured by a Pt/ZnO_*x*_/Pt memristor prepared in the laboratory. The results show that noise reduction effect can reach about 10%, while quantized CNN still achieves 91.97% of the recognition rate of the original image.

## Materials and Methods

### Device Fabrication and Operation

**FIGURE 1:**
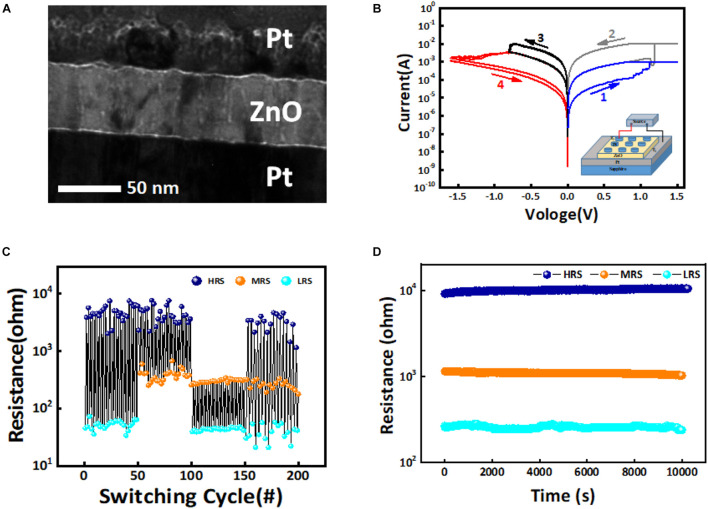
**(A)** Transmission electron microscope (TEM) image of the Pt/ZnO/Pt memristor. **(B)** Direct current (DC) current–voltage characteristics of the Pt/ZnO/Pt memristor showing three-state switching behavior. **(C,D)** Show the room-temperature endurance and retention performance of the device, respectively.

The ZnO-based memristors were fabricated by RF magnetron sputtering deposition of 50-nm ZnO_*x*_ thin film on commercial Pt/Ti/SiO_2_ wafers (HF-Keijing, Hefei, China), in a pure argon environment with 1.0-Pa environment pressure and 60-W AC radio frequency at room temperature for 5 min. Then 50-nm-thick round Pt electrodes with a diameter of 200 μm was deposited though E-beam deposition to form the Pt/ZnO/Pt structure. The transmission electron microscope (TEM) image of the Pt/ZnO/Pt memristor prepared is shown in Figure 1A. Applying sufficiently high voltage to the Pt/ZnO/Pt memristor electrode can drive the migration of oxygen anions or vacancies to form indentations, so that the memristor can undergo a resistance transformation phenomenon.

### Device Performance Evaluation

In general, memristors employ a conduction mechanism of conductive filaments (CFs), in which the conductive filaments can significantly regulate the resistance/conductance of the device through ion migration and solid-state redox reactions (Ambrogio et al., 2016; Chakrabarti et al., 2017; Hermann et al., 2020). In the direct current (DC) scanning voltage mode, the device exhibits obvious bipolar resistor switching behavior and a large memory window suitable for multi-stage modulation, as shown in Figure 1B. Three stable resistance states can be obtained by controlling the limiting current during the set process and the cutoff voltage during the reset process. We apply the scanning voltage from top electrode T_1_ to electrode T_2_ to the device. When the applied voltage rises from 0 to 0.9 V (scan 1 in Figure 1B, blue curve), the device will change from high resistance state (HRS) to middle resistance state (MRS). The corresponding resistance ranges are (970 to 2,564 Ω) and (119 to 223 Ω) for HRS and MRS, respectively. Similarly, scan 2 shows the current change from the MRS to the low resistance state (LRS, ∼71 to 83 Ω) when the applied voltage is increased to 1.2 V (gray curve). Scanning 3 and 4 show the current change curves of the device from LSR to MRS and from MRS to HRS when the voltage is applied from 0 to −0.7 and −1.5 V, respectively. Especially, the reset process for the device from MRS to HRS is different from the mutation of other phases. This slow change in resistance is conducive to selecting the appropriate programmed voltage to control the multiple conductance states of the memristor to achieve the image denoising based on memristor. As shown in Figure 1C, the MRS/LRS and HRS/MRS resistance conversion ratios taken in the experiment were all close to 10, and all resistance states could be programmed and read repeatedly. Meanwhile, the resistance value can be maintained at room temperature for a long time (Figure 1D).

**FIGURE 2:**
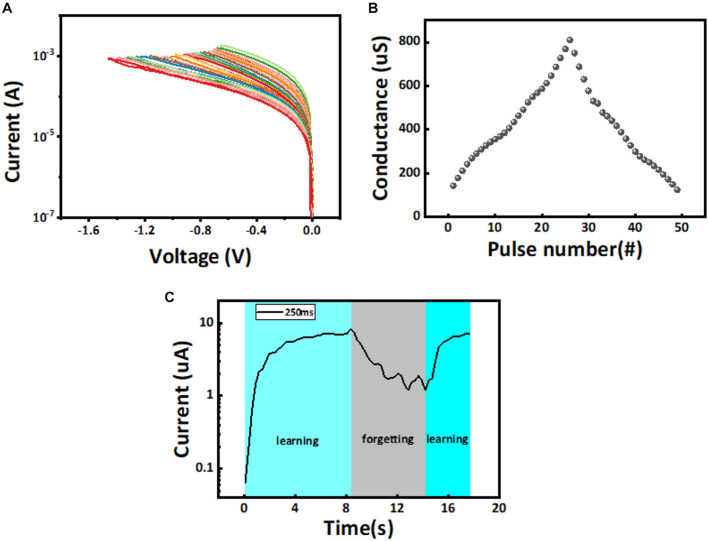
**(A)** Continuous regulation of the device current in the negatively biased reset processes. **(B)** Evolution of the device conductance as a function of the voltage pulse stressing numbers. All the voltage pulses show the same width of 200 ms and amplitude of 1.8 V. **(C)** The simulation of learning–forgetting–learning process based on long-term potentiation (LTP) with the positive voltage (pulse width of 200 ms, pulse interval of 50 ms, and amplitude of −0.4 V) and long-term depression (LTD) with the negative voltage (pulse width of 200 ms, pulse interval of 50 ms, and amplitude of −0.4 V) of memristor synapses.

Regulating the multiple conductance states of memristors can usually be achieved by controlling current compliance during set process or by changing cut-off voltage during reset process (Xue et al., 2019a,b). Since the positive feedback in the set process usually leads to the uncontrolled overgrowth of the CFs in the overshooting of device conduction, we adopt a relatively gentle reset process to modulate the evolution of the CFs (Chen et al., 2019). Note that the incorporation of transistor into the memristor crossbar array may also solve this problem (Hu et al., 2018). As shown in Figure 2A, in the DC scanning mode, we performed the reset operation on the Pt/ZnO/Pt device and applied a cutoff voltage from −0.6 to −1.5 V, and found that the device current decreased continuously. In addition, 26 continuously tunable conductance states were obtained by applying a train of voltage pulses with the same amplitude of 1.8 V and width of 200 ms to the electrode (Figure 2B). Based on the resistive memory performance aforementioned, the further investigation of the neurosynaptic bionics, which is generally evaluated by the dynamic weight changes, contain long-term potentiation (LTP) and long-term depression (LTD). As is shown in Figure 2C, the output current of memristor synapse increases first, then partially decreases, and finally rises rapidly in the process of learning–forgetting–learning (Wang et al., 2016; Zhang X. M. et al., 2020). This process is similar to that of human learning for the first time, which takes a lot of time. After a period of forgetting, the second learning can be learned quickly because part of the memory has already been acquired. The learning stage is the LTP process of applying positive pulse to the device. On the contrary, the forgetting stage is the LTD process of device with the negative pulses applied. The forgetting procedure observed herein is partially volatile with relatively lower current level of the device, which can be ascribed to the spontaneous recombination of the oxygen anions with the vacancies of the narrow filament formed in the device. Dimension variation or even dissolution of the filament may lead to decreasing of the device current, which is also originally small when compared with that of the device with strong filament. As such, the “learn–forget–learn” behavior is normally observed when the current level of the device is relatively low and associated with the weak filament.

## Results

### Image Denoising Based on Memristor

The device maintains LRS before the reset voltage increases from 0 to −0.7 V. However, between −0.7 and −0.8 V, we can see from Figure 1B that the device transition from LRS to MRS is not stable. From −0.8 to −1.5 V, the device will show a slow change of 26 conductance states during the transition from MRS to HRS. If the input voltage of the memristor is defined as −0 to −1.5 V, then the output states of the device, LRS, MRS, and HRS, can be defined as logical “0,” “1,” and “2,” respectively, in which the MRS and HRS will be divided into 26 logic states in the area [1,2]. According to this feature, we can map a picture with a pixel value between 0 and 255 to the voltage as the input of the memristor and then get a denoising picture of quantized by the multi-conductance memristor.

**FIGURE 3:**
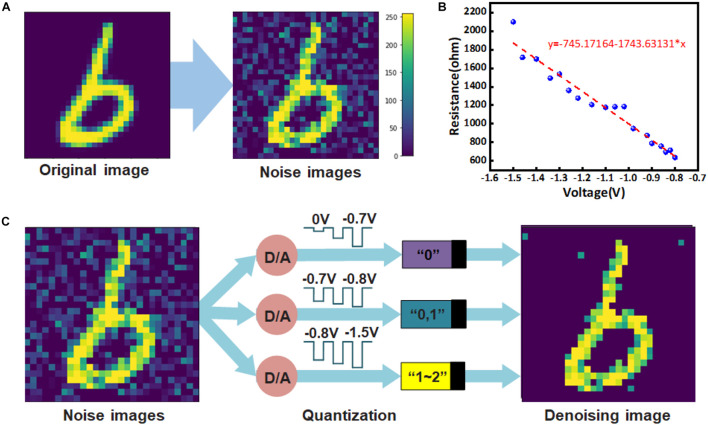
**(A)** An example to show that the original features of an image are reduced after the noise is added. The change in color from dark to light indicates a change in pixel value from 0 to 255. **(B)** The curve is obtained by linear fitting of the measured multi-resistance states of Pt/ZnO/Pt memristor as a function of voltage. **(C)** The process of image denoising by memristor.

The existence of noise not only seriously affects the quality of the image, but also hinders the reception of information. Especially in the field of artificial intelligence, the recognition and prediction of the machine is usually trained on the data set with obvious features and without interference factors, and its performance is often greatly reduced in the case when the input information contains noise (Andrews and Hunt, 1977; Chatterjee and Milanfar, 2010). As shown in Figure 3A, the image with added noise will not only weaken the feature pixel value, but also increases the original pixel with 0 value, thus, increasing the difficulty of prediction. Therefore, the key to image denoising lies in how to retain important feature information and remove noise information. In this work, the Gaussian white noise is generated and added to the pristine image with white Gaussian noise function imnoise (x, “Gaussian,” 0, σ^2^) based on the MATLAB platform. The variance σ^2^ uses the Randn function to generate randomly distributed values within [0–0.5], so the noise level is different for each image. For example, 124,800 variance σ^2^ were randomly generated within the range of [0–0.5] as the noise level to be added to each image in the training data set.

From the conductance distribution of the device, we found that if a picture with a pixel value between 0 and 255 is mapped to the reset voltage of 0 to −1.5 V, most of the noise pixels will be concentrated in the low voltage range of 0 to −0.7 V, then all the output of the device will be “0.” The intermediate pixel points usually cannot accurately locate whether they are noise or characteristic information, so they are distributed between −0.7 and −0.8 V. At this time, the output conductance state of the corresponding memristor will also have a certain randomness. The more obvious feature pixels will be between −0.8 and −1.5 V, and the input at this time will make the memristor show the characteristics of slowing changing conductance state. Therefore, we performed curve fitting for the multi-conductance states of the memristor based on Figure 2A, as shown in Figure 3B. The quantization method of prominent feature information will be determined by the linear function *y* = a + b*x* after fitting, with *a* = −745.17164 and *b* = −1,743.63131. The specific process of image quantization and noise reduction using the multi-resistance state of memristor is shown in Figure 3C. The image is converted from the pixel matrix of 0 to −1.5 V reset voltage matrix, which is used as the input of the memristor with the initial state of LRS. Where the input voltage between 0 and −0.7 V corresponds to a memristor output of “0,” this process is used to remove image noise. The input from −0.7 to −0.8 V corresponds to the output of the memristor, which is randomly assigned “0” or “1,” and this process retains weak characteristic information. The input from −0.8 to −1.5 V corresponds to the uniform distribution of the memristor output between “1” and “2,” and this process retains strong characteristic information. Therefore, after denoising the image by the memristor, it will be changed into a multi-conductance state matrix containing only 27 conductance states, so that the effect of image denoising can be achieved, and the original prominent feature information of the image can be retained. It is noteworthy that when convolution neural network is used to perform the recognition task for CIFAR images, contour feature extraction will be conducted. The value of the feature pixel is much larger than that of the background pixel. Therefore, the background of the noise image can still be quantized to 0 pixels in the process of image quantization, so as to achieve the effect of noise reduction for images without a clear background.

### Implement Weight Quantization Based on 26 Conductance States

**FIGURE 4:**
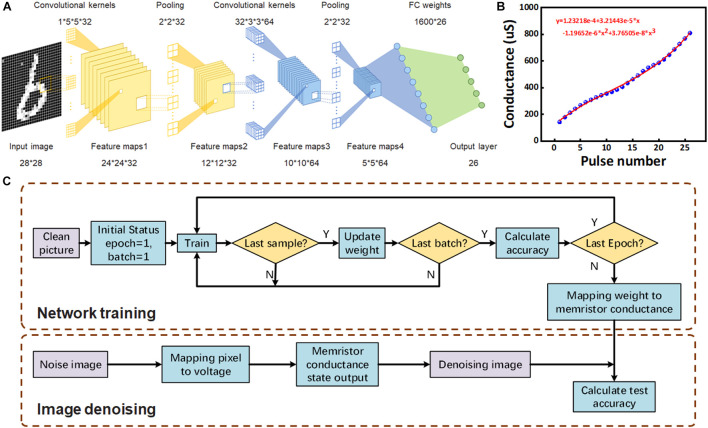
**(A)** CNN architecture for 26 handwritten letter recognition. **(B)** The function curve fitted with 26 conductance states regulated by pulse. **(C)** Schematic flowchart for the simulation of supervised learning with CNN for image denoising and recognition.

CNN is the most commonly used deep neural network for image recognition. In addition to the storage of a large number of continuous weight values, its computing process also requires a large number of convolution operations by the processor (Wang et al., 2019; Lin et al., 2020; Mukherjee et al., 2020; Liu and Yang, 2021). However, the processing efficiency of the computer is often unsatisfactory. In-memory computation of memristors can effectively improve the computational efficiency of neural networks. According to Figure 2B, 26 continuously adjustable conductance states were obtained by applying pulse voltage. We performed weight quantization operation for the CNN used in this paper, and handwritten letter recognition was done based on the quantized network. As shown in Figure 4A, the network consists of two convolutional layers, two pooling layers, and a full connection layer, which is a typical Lenet-5 structure (Le et al., 1989). The first convolution layer contains 32 5 × 5 convolution kernels, while the pooling layer is all 2 × 2 structure. The second convolution layer contains 2,048 3 × 3 convolution kernels. According to statistics, in terms of the number of weights, the first-layer convolution kernel only contains 800 weights, while the second-layer convolution kernel contains 18,432 weights, which accounts for about one-third of the total number of weights to be stored in the network. For the implementation of neural network hardware algorithm, such a large weight is difficult to be stored by accurate voltage regulation. In order to solve this problem, we need to quantify the second-layer convolution kernel weight of the network, and the device prepared above is suitable for realizing this process. Figure 4B shows that the device has 26 stable and continuous conductance states under the pulse voltage. The relationship between the number of pulses and the conductance is obtained by means of polynomial fitting, and the mapping relationship between the weights of second-layer convolution kernel and the conductance of the device is constructed based on this. The optimized fitting function is obtained as *y* = a + b*x* + c*x*^2^+d*x*^3^, with *a* = 1.23218 × 10^–4^, *b* = 3.21443 × 10^–5^, *c* = −1.19652 × 10^–6^, and *d* = 3.76505 × 10^–8^. In this process, we divide the original weight distribution into 26 intervals, so all the weights will fall within a corresponding interval. Each interval corresponds to a conductance state, and the quantized weight will be mapped to 26 conductance states. According to the network training results in the experiment, we found that the weights were continuously distributed in [−1.30, 0.83]. According to the fitting results in Figure 4B, [−1, 1] is divided into 26 regions, and the network weight can be quantized into 26 discrete memristor weight values in [−1, 1]. After quantization, the weight matrix only needs to design 26 input pulses to realize the weight storage, which improves the fault tolerance rate of the hardware realization of weight storage. Backpropagation (BP) algorithm was used to demonstrate and simulate supervised learning, including the training of the above CNN weights, the corresponding memristor conductance update, and the test of denoising images (Figure 4C). The general process is mainly divided into the stage of network training and the stage of image denoising. In the stage of network training, the original image data set is used to train the network weight, and the second-layer convolution kernel weight after training is quantized. In the stage of image denoising, we used the multi-conductance state property to optimize noise image and test optimization results through the trained CNN.

Based on the above method, we use the multi-conductance states of memristor to achieve denoising and recognition of noisy images. The dataset used in the network simulation is taken from the National Institute of Standards and Technology (NIST) Special Database, which is an extension of MNIST to handwritten letters called Extended MNIST (EMNIST). For the 26 English letters, the database consists of 124,800 training samples, and each letter contains 4,800 upper- and lowercase samples. There are 20,800 test samples, and each letter contains 800 upper- and lowercase samples. In the network training stage, the original data samples were used, while in the test stage, the original samples, noise samples, and denoising samples were recognized, and the final accuracy was 96.48, 64.54, and 74.73%, respectively. After that, we use 26 conductance states of Pt/ZnO/Pt memristor to carry out weight quantization on the second layer convolution kernel of CNN and test the network. The results confirm that the quantized memristor neural network can still achieve the recognition accuracy of 91.97% on the original data (Table 1). At the same time, Table 1 also indicates that the proposed image denoising scheme based on Pt/ZnO/Pt memristor is feasible.

**TABLE 1:** The results of three samples were tested on unquantized and quantized CNN.

Dataset	Recognition accuracy	
	
	Unquantized	Quantized
Original image	96.48%	91.97%
Noise image	64.54%	50.37%
Denoise image	74.73%	62.34%

**FIGURE 5:**
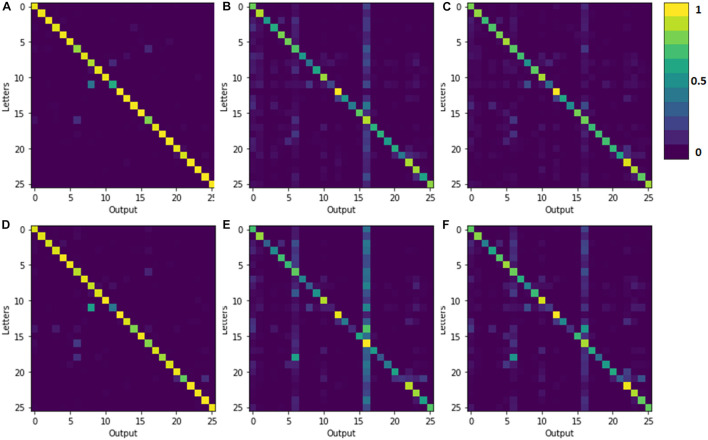
The confusion matrix obtained after testing the samples. **(A,D)** The initial samples, **(B,E)** the noise samples, and **(C,F)** the denoising samples for the unquantized CNN and the quantized CNN, respectively. The ordinate denotes 26 input testing letters, and the abscissa denotes the output and recognition results.

In order to further explain the experimental results, we extracted the confusion matrices obtained by the network under different conditions for different test samples (Figure 5). By comparing Figures 5A,D, it can be clearly seen that the network can still maintain a good recognition rate after quantifying one of the layers of CNN. Figures 5B,C,E,F all indicate that the device prepared can effectively remove part of the noise in the image.

It is noteworthy that the inherent noise of the memristor is indeed a critical issue that influences the practical application of this novel device technology. To avoid the fluctuation of device conductances that may deteriorate the overall image processing performance, a good practice is to use conductance states that do not overlap with each other during repeated switching operations. There are approaches to achieve such a target, and we are also making efforts toward the goal. Nevertheless, the main purpose of this work is to demonstrate that memristors with multi-level switching characteristics can theoretically be used for feature preservation and noise reduction in image-processing applications.

## Conclusion

In this work, we demonstrate a simple image denoising method based on 26 conductance states of Pt/ZnO/Pt memristor and further construct a convolutional neural network for image denoising and recognition. By analyzing the conductance distribution characteristics of Pt/ZnO/Pt memristor, the mapping relationship between device conductance and image pixel value was constructed. The image was taken as the input of the memristor in the form of voltage, and the corresponding output conductance state was taken as a result of image quantization to realize the denoising function. Moreover, the weight of CNN is quantized by the conductance distribution measured by the pulse voltage, and the weight quantization rules are designed to implement the memristor neural network effectively. In addition to the intrinsic storage capability of the memristor for in-memory computing, the multi-conductance states property cannot only accurately quantify the weight of neural network but also play a role in image denoising and feature screening.

## Data Availability Statement

The original contributions presented in the study are included in the article/supplementary material, further inquiries can be directed to the corresponding authors.

## Author Contributions

YZ and ZW designed the image denoising scheme, CNN structure, and the map of weight and conductance. QC prepared the device and performed the performance tests. QC and SL provided help for the design of the simulation method. ZW carried out the simulation work and experiments. ZW, ZG, and GL were involved in the writing and editing of the manuscript. GL, QC, YZ, and PW oversaw the project. All authors discussed the results.

## Conflict of Interest

The authors declare that the research was conducted in the absence of any commercial or financial relationships that could be construed as a potential conflict of interest.

## Publisher’s Note

All claims expressed in this article are solely those of the authors and do not necessarily represent those of their affiliated organizations, or those of the publisher, the editors and the reviewers. Any product that may be evaluated in this article, or claim that may be made by its manufacturer, is not guaranteed or endorsed by the publisher.
